# Traumatic Failure of Primary Latarjet Revised With Distal Tibial Allograft Reconstruction: A Case Report

**DOI:** 10.7759/cureus.108910

**Published:** 2026-05-15

**Authors:** Ryan M Marra, Christopher McDowell, Jacob Turnbull

**Affiliations:** 1 Orthopedic Surgery, Duquesne University Nasuti College of Osteopathic Medicine, Pittsburgh, USA; 2 Orthopedics, Orthopedics East and Sports Medicine Center, Greenville, USA; 3 Community Medicine, Duquesne University Nasuti College of Osteopathic Medicine, Pittsburgh, USA

**Keywords:** anterior shoulder instability, coracoid process, laterjet repair, recurrent shoulder instability, shoulder trauma surgeon

## Abstract

Recurrent anterior shoulder instability in young, high-demand patients is frequently compounded by significant glenoid bone loss, a known risk factor for failure of isolated soft-tissue stabilization. The Latarjet procedure has demonstrated reliable outcomes in restoring stability in this population; however, traumatic postoperative complications such as coracoid graft fracture and hardware failure are rare and pose substantial challenges in revision management. Optimal strategies for addressing failed coracoid transfer remain incompletely defined. In this case report, a case of traumatic failure of a primary Latarjet procedure is reported and distal tibial allograft (DTA) reconstruction as a biologically and anatomically favorable revision technique is evaluated. An adolescent male patient with recurrent anterior shoulder instability and critical anterior glenoid bone loss underwent primary Latarjet stabilization for a chronic bony Bankart lesion. At three months postoperatively, the patient sustained a high-energy traumatic reinjury. Advanced imaging demonstrated coracoid graft fracture with associated hardware failure. Revision surgery was performed with the removal of failed hardware and reconstruction of the anterior glenoid using a distal tibial allograft, selected for its congruent articular surface and structural compatibility with native glenoid anatomy. A standardized postoperative rehabilitation protocol was implemented. Clinical and radiographic outcomes were assessed longitudinally. The patient experienced an uncomplicated postoperative course with progressive functional recovery. Serial imaging confirmed appropriate graft positioning, stability, and osseous incorporation without evidence of resorption or complication. At final follow-up, the patient demonstrated near-complete restoration of range of motion and strength, with no recurrent instability and successful return to unrestricted activity. Distal tibial allograft reconstruction represents a robust and anatomically restorative option for the revision of failed Latarjet procedures following traumatic graft compromise. This technique offers several advantages, including preservation of native glenoid contour, restoration of the articular surface, and avoidance of donor-site morbidity associated with autograft harvest. In young, high-demand patients, DTA reconstruction may provide reliable stability and favorable functional outcomes in complex revision settings. Further investigation is warranted to establish long-term outcomes and comparative efficacy relative to alternative revision strategies.

## Introduction

Recurrent anterior shoulder instability in young, high-demand patients is frequently associated with anterior glenoid bone loss, which significantly increases the risk of failure following isolated soft-tissue stabilization procedures [1]. The Latarjet procedure is a well-established surgical intervention for patients with well-established anterior glenoid defects and has demonstrated lower recurrence rates compared with soft-tissue repair alone [2].

Although primary Latarjet outcomes are generally favorable, traumatic postoperative complications, such as coracoid graft fracture and hardware failure, are rare but can present complex clinical challenges [3]. The management of failed coracoid transfer procedures remains technically demanding, and consensus regarding optimal revision strategies is limited [4].

This case report describes a traumatic failure of a primary Latarjet procedure in a young patient and highlights fresh distal tibial allograft reconstruction as a revision technique aimed at restoring native glenoid anatomy and stability. This approach has been previously described in the literature [5-7]; however, additional case series and comparative studies are needed to better evaluate its effectiveness across diverse patient populations and its long-term reliability. This report offers a unique perspective as patients undergoing severe traumatic displacement of the coracoid graft with additional need to return to high-level military activity are underrepresented in the literature. 

## Case presentation

**Figure 1:**
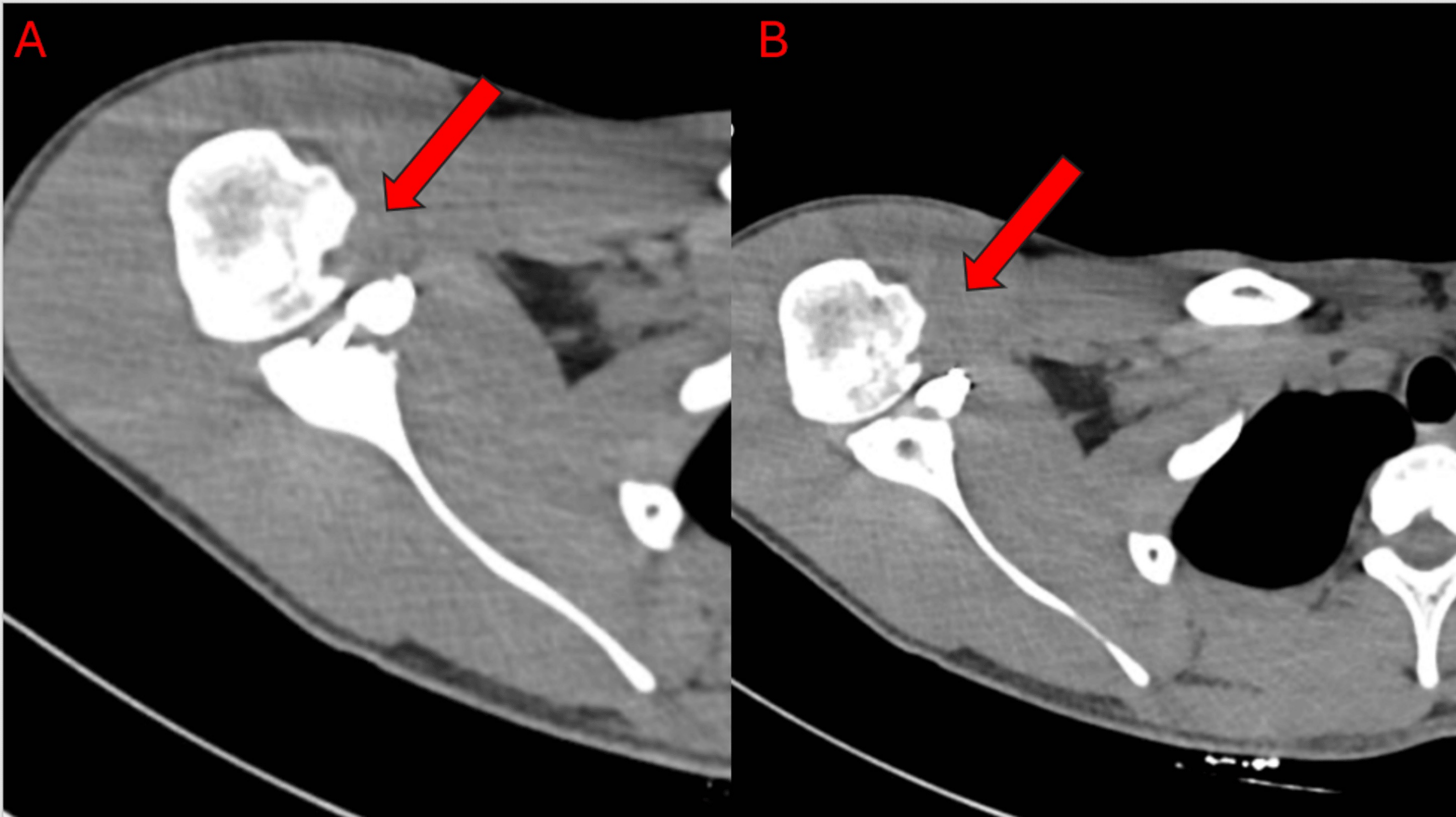
MRI of the right shoulder demonstrating significant anterior glenoid bone loss associated with recurrent anterior shoulder dislocations. Axial MRI images progressing from superior (A) to inferior (B) levels demonstrating a concomitant superior labral tear in the anterior-posterior plane lesion, Hill-Sachs deformity, and bony Bankart lesion associated with recurrent anterior shoulder instability.

An adolescent male with a history of recurrent anterior shoulder instability presented with persistent instability and significant anterior glenoid bone loss. Preoperative imaging demonstrated a chronic bony Bankart lesion (Figure 1), and the patient underwent primary open reduction and internal fixation with coracoid transfer via the Latarjet procedure.

The initial postoperative course was uncomplicated. However, approximately three months following surgery, the patient sustained a high-energy traumatic injury after being struck by a motor vehicle while riding a bicycle. He subsequently reported acute-onset shoulder pain and instability.

**Figure 2:**
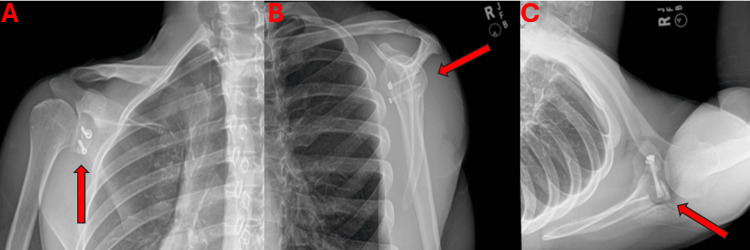
Radiographic images of the right shoulder obtained following traumatic injury demonstrating coracoid graft displacement and hardware failure after primary Latarjet procedure. Pre-revision radiographic views of the right shoulder demonstrating posterior displacement of the coracoid graft with hardware failure, including a bent proximal screw and posterior migration of the distal screw, shown in anterior (A), posterior (B), and axial (C) orientations.

Radiographic evaluation revealed a fracture and posterior displacement of the coracoid graft, with associated hardware failure characterized by screw bending and loosening (Figure 2). Given the extent of graft disruption and instability, revision surgical intervention was indicated.

**Figure 3:**
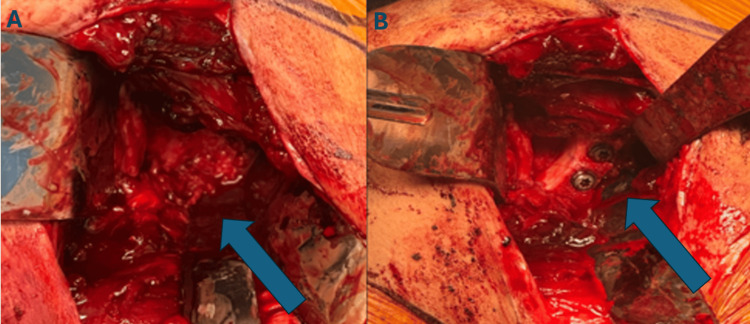
Intraoperative images obtained during revision anterior glenoid reconstruction using distal tibial allograft. Intraoperative images obtained during revision surgery demonstrating the fractured coracoid graft and failed fixation construct (A), followed by placement and fixation of the distal tibial allograft for anterior glenoid reconstruction (B).

The patient underwent removal of failed hardware and reconstruction of the anterior glenoid using a distal tibial allograft. The allograft was selected for its anatomic curvature and cartilage congruency with the native glenoid surface. The graft was contoured to match the defect and secured to the anterior glenoid using screw fixation to restore stability and joint congruity (Figure 3).

**Figure 4:**
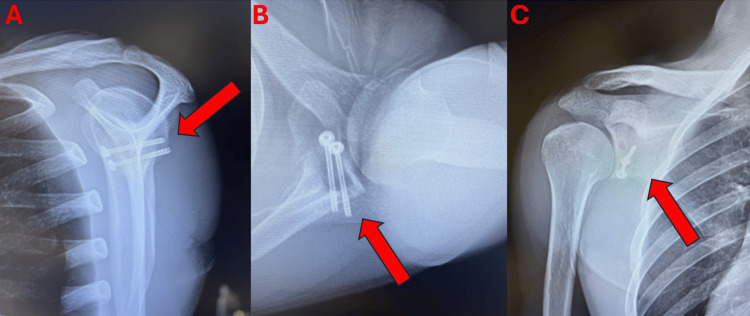
Postoperative radiographs of the right shoulder following revision anterior glenoid reconstruction with distal tibial allograft. Postoperative radiographic views of the right shoulder following distal tibial allograft reconstruction for revision anterior glenoid stabilization, demonstrating graft positioning and fixation in the posterior (A), axial (B), and anterior (C) orientations.

Postoperatively, the patient was placed on a structured rehabilitation protocol. Serial clinical evaluations demonstrated progressive improvement in range of motion and strength. At final follow-up, the patient achieved near-full shoulder function without recurrent instability and successfully returned to unrestricted physical activity. Radiographic imaging confirmed stable graft fixation and evidence of osseous incorporation without complication (Figure 4). A limitation of this report is the absence of standardized postoperative functional outcome measures such as the American Shoulder and Elbow Surgeons (ASES) or Constant scores, which would have provided a more objective assessment of clinical recovery.

## Discussion

The failure of the Latarjet procedure is uncommon but presents significant clinical challenges when it occurs, particularly in the setting of traumatic graft disruption [3]. Revision strategies must address both mechanical instability and restoration of glenoid bone stock while minimizing additional morbidity [4].

Distal tibial allograft reconstruction has gained increasing attention as a viable option for glenoid reconstruction. The distal tibia provides a dense osteochondral graft with curvature closely matching that of the native glenoid, allowing for an anatomic restoration of the articular surface [8]. This characteristic is particularly advantageous compared to other graft options, as it preserves joint congruity and may reduce the risk of postoperative arthrosis [9].

Additionally, the use of allograft eliminates donor-site morbidity associated with autograft harvest and avoids the technical limitations of repeat coracoid transfer, which may not be feasible in the setting of prior graft failure. Biomechanical studies have demonstrated that distal tibial allograft reconstruction can effectively restore glenoid surface area and stability [5-7].

The etiology of Latarjet failure is multifactorial and may include technical errors, graft malposition, nonunion, or, as in this case, high-energy trauma [3]. Management must therefore be individualized based on patient factors, extent of bone loss, and the integrity of the remaining structures. In the present case, failure was most likely related to the high-energy traumatic event rather than an isolated technical error. The patient had an initially uncomplicated postoperative course without evidence of instability or hardware complications prior to the collision. Although technical factors may contribute to Latarjet failure, the severity of the trauma likely exceeded the tolerance of the healing construct, resulting in graft fracture and hardware failure.

This case supports the growing body of evidence suggesting that distal tibial allograft reconstruction is a reliable and effective revision strategy [5-7]. The favorable clinical and radiographic outcomes observed highlight its role in restoring stability and function in complex cases of anterior shoulder instability.

Alternative revision options for anterior shoulder instability include iliac crest autograft (Eden-Hybinette procedure), distal clavicle autograft, and structural femoral head allograft [10-12]. Iliac crest autograft remains a commonly utilized technique because it provides substantial structural support and reliable restoration of glenoid bone stock; however, donor-site morbidity and lack of an articular cartilage surface remain important limitations [10]. Distal clavicle autograft offers a local graft source with preserved osteochondral characteristics, though graft size and availability may limit its application in larger defects [11]. Structural allograft techniques, including femoral head and distal tibial allografts, avoid donor-site morbidity while permitting anatomic restoration of the glenoid articular surface [12]. Compared with these alternatives, the distal tibial allograft provides dense osteochondral architecture with curvature closely resembling the native glenoid, which may improve joint congruity and reduce the risk of postoperative arthrosis [8,9]. Nevertheless, long-term comparative outcome studies remain limited, and no clear consensus currently exists regarding the optimal graft choice in revision settings.

This report has several limitations. First, the follow-up period was limited to six months after the revision procedure, restricting the assessment of long-term graft incorporation, recurrent instability, and development of glenohumeral arthrosis. Second, as a single-case report, the findings may not be generalizable across broader patient populations or alternative mechanisms of failure. Larger comparative studies with longer follow-up are needed to better define the durability and long-term clinical outcomes of distal tibial allograft reconstruction in revision anterior shoulder instability.

## Conclusions

Traumatic failure of a primary Latarjet procedure is a rare but challenging complication that requires individualized surgical management. Distal tibial allograft reconstruction may represent a useful revision option by restoring glenoid anatomy and shoulder stability while avoiding donor-site morbidity associated with autograft harvest.

In this case, distal tibial allograft reconstruction resulted in favorable short-term clinical and radiographic outcomes following failed Latarjet fixation. However, additional studies with larger patient populations and longer follow-up are needed to better define the durability, long-term outcomes, and optimal role of this technique in revision shoulder instability surgery.
